# Feature Subset Selection for Cancer Classification Using Weight Local Modularity

**DOI:** 10.1038/srep34759

**Published:** 2016-10-05

**Authors:** Guodong Zhao, Yan Wu

**Affiliations:** 1 School of Electronics and Information Engineering, Tongji University, Shanghai 201804, China

## Abstract

Microarray is recently becoming an important tool for profiling the global gene expression patterns of tissues. Gene selection is a popular technology for cancer classification that aims to identify a small number of informative genes from thousands of genes that may contribute to the occurrence of cancers to obtain a high predictive accuracy. This technique has been extensively studied in recent years. This study develops a novel feature selection (FS) method for gene subset selection by utilizing the Weight Local Modularity (*WLM*) in a complex network, called the *WLMGS*. In the proposed method, the discriminative power of gene subset is evaluated by using the weight local modularity of a weighted sample graph in the gene subset where the intra-class distance is small and the inter-class distance is large. A higher local modularity of the gene subset corresponds to a greater discriminative of the gene subset. With the use of forward search strategy, a more informative gene subset as a group can be selected for the classification process. Computational experiments show that the proposed algorithm can select a small subset of the predictive gene as a group while preserving classification accuracy.

Gene expression microarray dataset technology plays a crucial role in helping researchers analyze thousands of genes simultaneously to assess the pathological diagnosis and classification of cancer diseases^1^. The gene selection from gene expression data is challenging because of properties such as small sample size, large dimensions, and high noise. Clinical diagnoses require the selection of a small predictive subset of biologically relevant genes with a high classification accuracy for cancers.

In recent years, different strategies have been proposed for feature selection, such as filter^2^, wrapper^3^,^4^, embedded^5^, and more recently, ensemble techniques^6^. In filter approaches, gene selection is dependent not on the classification algorithm but on a criterion that assesses the relevance or importance of each gene for class label discrimination on the basis of the generic characteristics of the data. Wrapper approaches are tightly coupled with specific learning algorithms to evaluate the generated subset of genes every time it is used and to achieve the best prediction performance for a special learning model^7^. However, wrapper approaches are more computationally intensive than filter approaches. Nonetheless, wrapper method is generally considered superior over other filters in terms of performance. Consequently, wrapper methods are often intractable for large -scale problems, particularly for microarray analysis^3^. Embedded techniques, search for an optimal subset of features depending on the classifier construction, which can be seen as a search in the combined space of feature subsets and hypotheses. Similar to wrapper approaches, embedded approaches are specific to a given learning algorithm, but the computational time is smaller compared to the wrapper methods^5^. Ensemble techniques have been proposed to cope with the instability issues observed in many techniques for FS when small perturbations in the training set occur. These methods are based on different subsampling strategies. A particular FS method is run on a number of subsamples and the obtained features are merged into a more stable subset. To date, filter methods are widely investigated by numerous researchers because of its simplicity and efficiency.

## Related Work

Owing to the importance of gene selection in the analysis of the microarray dataset and the diagnosis of cancer, various techniques for gene selection problems have been proposed.

Because of the high dimensionality of most microarray analyses, fast and efficient gene selection techniques such as univariate filter methods^8^,^9^,^10^ have gained more attention. Most filter methods consider the problem of FS to be a ranking problem. The solution is provided by selecting the top scoring features/genes while the rest are discarded. Scoring functions represent the core of ranking methods and are used to assign a relevance index to each feature/gene. The scoring functions mainly include the Z-score^11^ and Welch t-test^12^ from the t-test family, the Bayesian t-test^13^ from the Bayesian scoring family, and the Info gain^14^ method from the theory-based scoring family. However, the filter-ranking methods ignore the correlations among gene subset, so the selected gene subset may contain redundant information. Thus, multivariate filter techniques have been proposed by researchers to capture the correlations between genes. Some of these filter techniques are the correlation-based feature selection (CFS)^15^, the Markov blanket filter method^16^ and the mutual information (MI) based methods, *e.g.* mRMR^17^, MIFS^18^, MIFS_U^19^, and CMIM^20^.

In recent years, the metaheuristic technique, which is a type of wrapper technique, has gained extensive attention and has been proven to be one of the best -performing techniques used in solving gene selection problems^21^,^22^. Genetic algorithms (GAs) are generally used as the search engine for feature subsets combined with classification methods. Some examples of GAs are the estimation of distribution algorithm (EDA) with SVM^23^,^24^,^25^, the genetic algorithms support vector machine (GA-SVM)^26^, and the K nearest neighbors/genetic algorithms (KNN/GA)^27^.

However, most of the existing methods, such as the mutual information based methods^17^,^18^,^19^,^20^, only choose the strong genes in the target class but ignore the weak genes which possess a strong discriminatory power as a group but are weak as individuals^3^.

Over the past few decades, complex network theories have been used in different areas such as biological, social, technological, and information networks. In this present study, a novel method is proposed to search for the ‘weak’ genes by using the sequential forward search strategy. In the proposed method, an efficient discrimination evaluation criterion of a gene subset as a group is presented based on the weight local modularity (WLM) in a complex network. This method employs the advantages of the weight local modularity which most networks are composed of. The WLM are communities or groups within which the networks have a locally small distance between the nodes, but have a relatively large distance between the various communities^28^. By constructing the weighted sample graph (WSG) in a gene subset, a large weight local modularity value means that the samples in the gene subset are easily separated locally, and that the gene subset is more informative for classification. Therefore, the proposed method has the capability to select for an optimal gene subset with a stronger discriminative power as a group. The effectiveness of method in this present study is validated by conducting experiments on several publicly available microarray datasets. The proposed method performs well on the gene selection and the cancer classification accuracy.

## Results

In this section, the experimental results and analysis of *WLMGS* on several public microarray datasets are presented. The proposed algorithms are programmed in the Matlab 2012b environment, and the simulations are performed with the use of an Intel Core i3-2310M-2.1 GHz CPU having 2 GB of RAM. The nearest neighborhood classifier (**1NN**) together with the Euclidean distance and support vector machine (**SVM)** classifiers with C = 100 and RBF kernel are utilized to assess the generated solutions.

In order to avoid the selection bias^29^, a 10-fold cross-validation over each dataset is performed in the genes section, wherein gene subsets are selected from the training instances (90%), and then, the accuracy is estimated over the test instances (10%). This process is performed 10 times. The final gene subset ***gs*** is composed of more frequent genes in the ten selected subsets. The precision in this present work is of 10 times accuracy average. Five filter methods and two wrapped methods are compared with the method used in this present study, and these include the CMQFS^30^, the mRMR, the MIFS-U, the CMIM, the Relief^31^, the SVMRFE^4^, and the KNNFS^32^ methods.

### Datasets

To validate the effectiveness of the method of this present study, several experiments were performed on some well-known public gene microarray datasets with a high dimensionality and small sample size. These datasets are downloaded from http://csse.szu.edu.cn/staff/zhuzx/Datasets.html and from^33^,^34^,^35^. A summary about these datasets is provided in Table 1. Before conducting the experiments, each represented gene was normalized, that is, its mean and standard deviation were set to zero and one, respectively.

In mutual information computations, the continuous features were discretized to nine discrete levels as in ref. 36 and 37. The feature values were converted to values between *μ*−*σ*/2 and *μ* + *σ*/2 to 0, the four intervals of size *σ* to the right of *μ* + *σ*/2 to discrete levels from 1 to 4, and the four intervals of size *σ* to the left of *μ*−*σ*/2 to discrete levels from −1 to −4. Very large positive or small negative feature values are truncated and discretized to ±4 appropriately. In this paper, the FEAST tool^38^ is used to calculate for the mutual information (MI) and the conditional mutual information (CMI).

### Computational results

**Figure 1:**
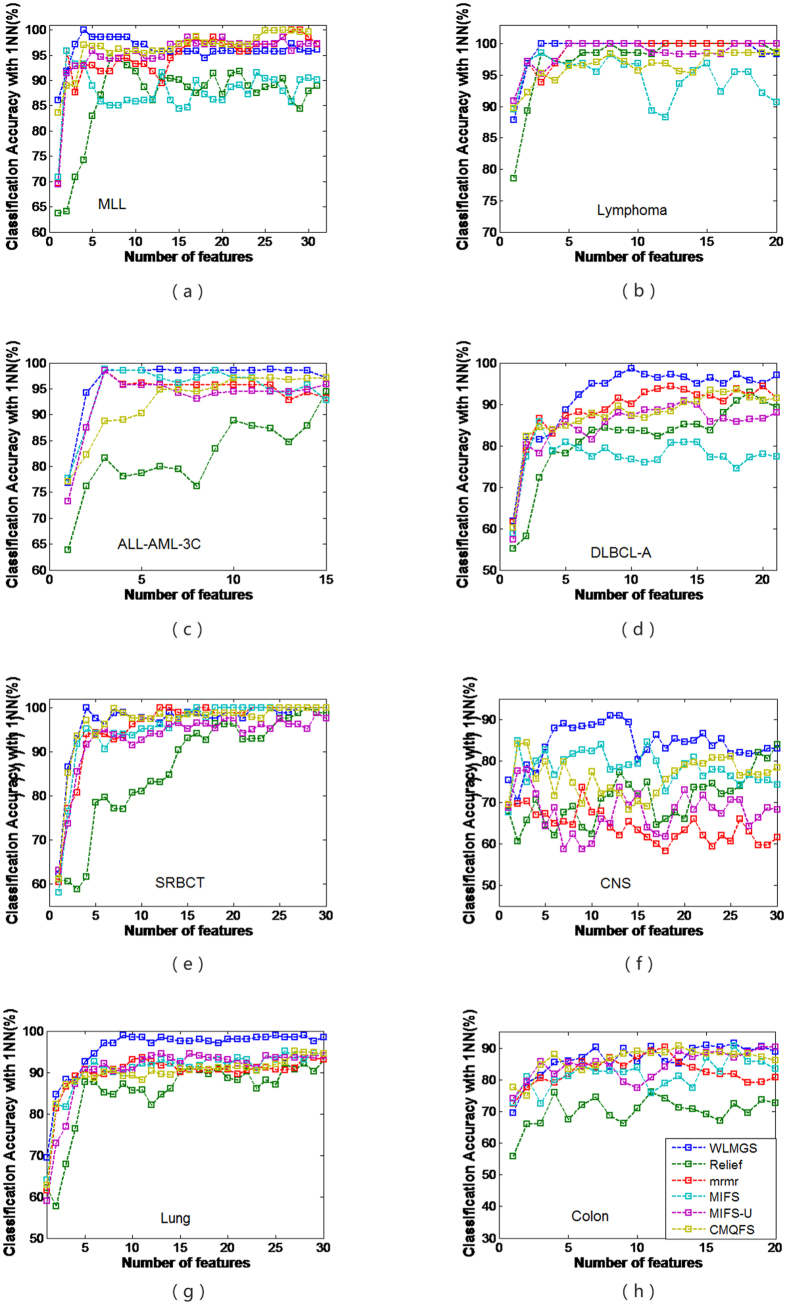
The average classification accuracy using 1NN classifier with respect to the subset of s features selected by different filter methods. For different methods, (**a**) is the classification accuracy in data MLL, (**b**) is the classification accuracy in data Lymphoma, (**c**) is the classification accuracy in data ALL-AML-3c, (**d**) is the classification accuracy in data DLBCL-A, (**e**) is the classification accuracy in data SRBCT, (**f**) is the classification accuracy in data CNS, (**g**) is the classification accuracy in data Lung, (**h**) is the classification accuracy in data Colon.

**Figure 2:**
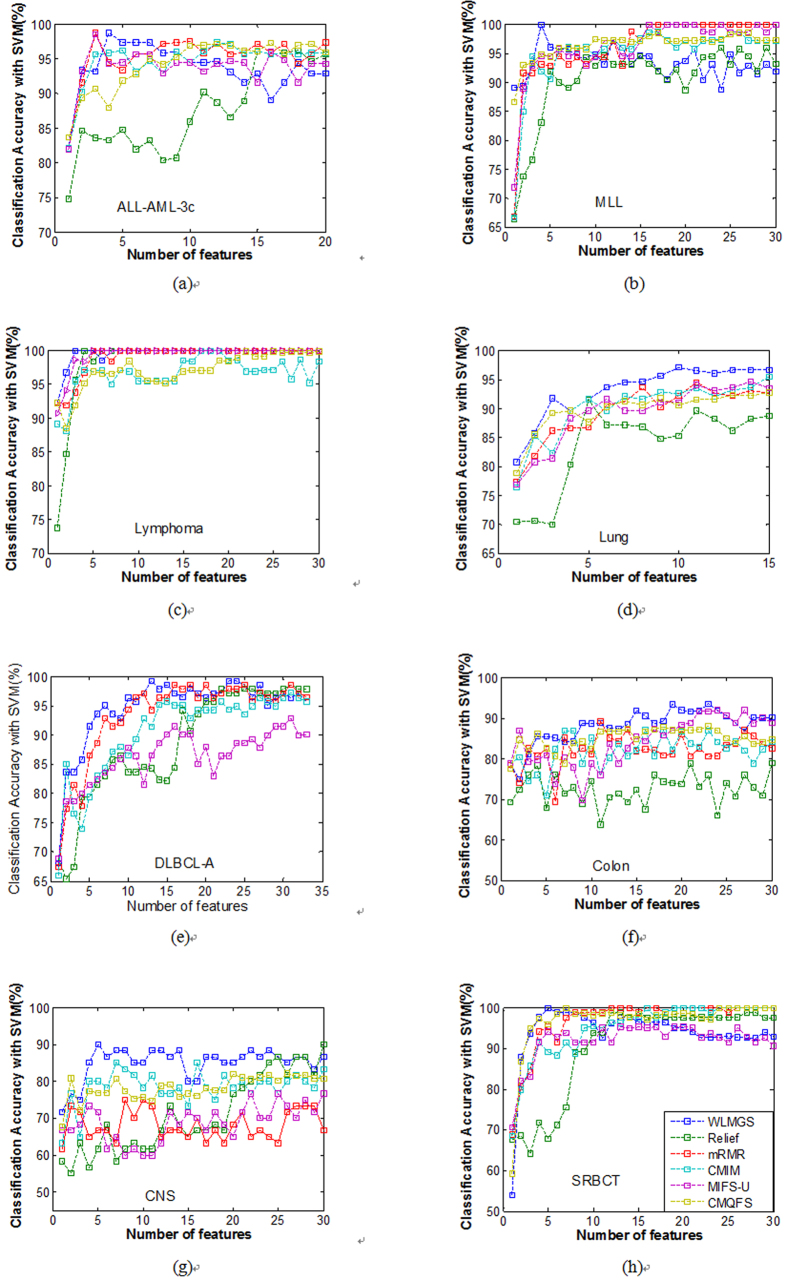
The average classification accuracy using SVM classifier with respect to the subset of s features selected by different filter methods. For different methods, (**a**) is the classification accuracy in data ALL-AML-3c, (**b**) is the classification accuracy in data MLL, (**c**) is the classification accuracy in data Lymphoma, (**d**) is the classification accuracy in data Lung, (**e**) is the classification accuracy in data DLBCL-A, (**f**) is the classification accuracy in data Colon, (**g**) is the classification accuracy in data CNS, (**f**) is the classification accuracy in data SRBCT.

In this present study, the related parameters *λ*, *θ* are set to 15 and 0.02, respectively. Tables 2 and 3 summarize the best classification accuracy in **1NN** and **SVM** classifiers under the selected genes, respectively, when compared with the five filter methods. Figures 1 and 2 are the average classification accuracies using the **1NN** and **SVM** classifiers at different number of genes selected by different methods, respectively. |#G| presents the number of selected genes when the best average accuracy is achieved. ACC is the best average accuracy, because it possesses a 10 times accuracy rate. The best average results are shown in bold. The improved performance of the method used in this present study is reached by using a different parameter *k*. For the filter methods in Tables 2 and 3, it is observed that the method used in this study outperforms the other filter methods, because it reaches a higher classification accuracy for the **1NN** and **SVM** classifiers in most cases.

For the **ALL-AML-3C** dataset, the method (*WLMGS*) in this present study obtains a good performance: 98.57% (1NN) and 98.75% (SVM), which are both higher compared to the *CMIM, mRMR, MIFS_U, and CMQFS,* although the 3 genes in the 1NN and the 4 genes in the SVM are selected.

For the **DLBCL_A** dataset, the algorithm in this study achieves a better prediction accuracy of 98.62%, with an average of only 10 genes in 1NN, and an accuracy of 99.28% with an average of only 13 genes in the SVM. However, the best algorithm, as compared with the other methods is the *mRMR,* which selects 22 genes to gain a prediction accuracy of 95.71% in 1NN, and a 98.66% prediction accuracy in SVM.

For the **SRBCT** dataset, the **MLL** dataset and the **Lymphoma** dataset, in **1NN (SVM)**, the method in this present study obtains perfect prediction results 100% (100%) with only an average of 4 (5) genes, an average of 4 (4) genes, and an average of 3 (3) genes respectively. These results are compared with the best results of *mRMR*: 100% with an average of 12 (12) genes, 100% with an average of 28 (16) genes, and 100% with an average of 5 (5) genes, respectively. It can be seen clearly that for these datasets, the *mRMR* algorithm produces better results as compared to the other methods. The key reason is that *mRMR* not only considers the relevance of genes, but it also considers the redundancy between genes. However, the *mRMR* method only measures the quantity of *irrelevant redundancy* (*IR*)^30^, but does not deal with its *relevant redundancy* (*RR*)^30^. This can cause a problem since this method chooses some irrelevant variables prematurely, and is delayed in picking out some useful variables^39^,^40^. Therefore, the best performance only can be obtained by using more genes. In this present study’s method, is used to measure the *relevant independency* among gene subset, which is helpful for classification*WLM*^*s*^. Therefore, all these useful genes, including the power genes as individuals, and the ‘power’ genes as a group are explored, and this contributes in gaining the best results by using fewer genes. The experiment results in this present study are perfectly consistent with the stated facts and confirm the effectiveness of this study’s approach.

In the **CNS** dataset, the average number of selected genes in this present study’s method is larger than the CMIM in **1NN** and **SVM**. However, the best results of this study are higher than CMIM.

In the **Lung** dataset, there are 139 samples in the first class and only 6 samples in the third class. For the imbalanced dataset, the method of this study achieves a higher accuracy of 99.02%, with an average of 9 genes in **1NN,** and 97.07% with an average of 10 genes in **SVM**. This is significantly superior compared to the other methods.

In the **Colon** dataset, this study’s method performs better than the other algorithms, although the number of selected genes of this study’s method is larger.

To further strengthen the efficiency of the method in this study, the **1NN** and **SVM** classification results with a different number of selected genes for all the methods are shown in Figs 1 and 2, respectively. Therefore, it can be concluded from Figs 1 and 2 that this study’s method achieves better results with fewer selected genes in **1NN** and **SVM** compared to other methods in most cases. These findings are identical with the results in Tables 2 and 3.

Furthermore, the results in this study are validated in Tables 4 and 5 by conducting a statistical paired samples one-tailed *test.* This statistical test is used to verify whether there is any significant difference in the accuracy and the number of selected genes by using a significance interval of 95% (*a* = 0.05). The results of this study show that all the *p*-values obtained are less than 0.05. This means that there is a significant difference in the accuracy and the number of selected genes in the method used in this study as compared to the other methods on all datasets, respectively. It can be concluded therefore that this study’s proposed method significantly outperforms the other algorithms.

**Figure 3:**
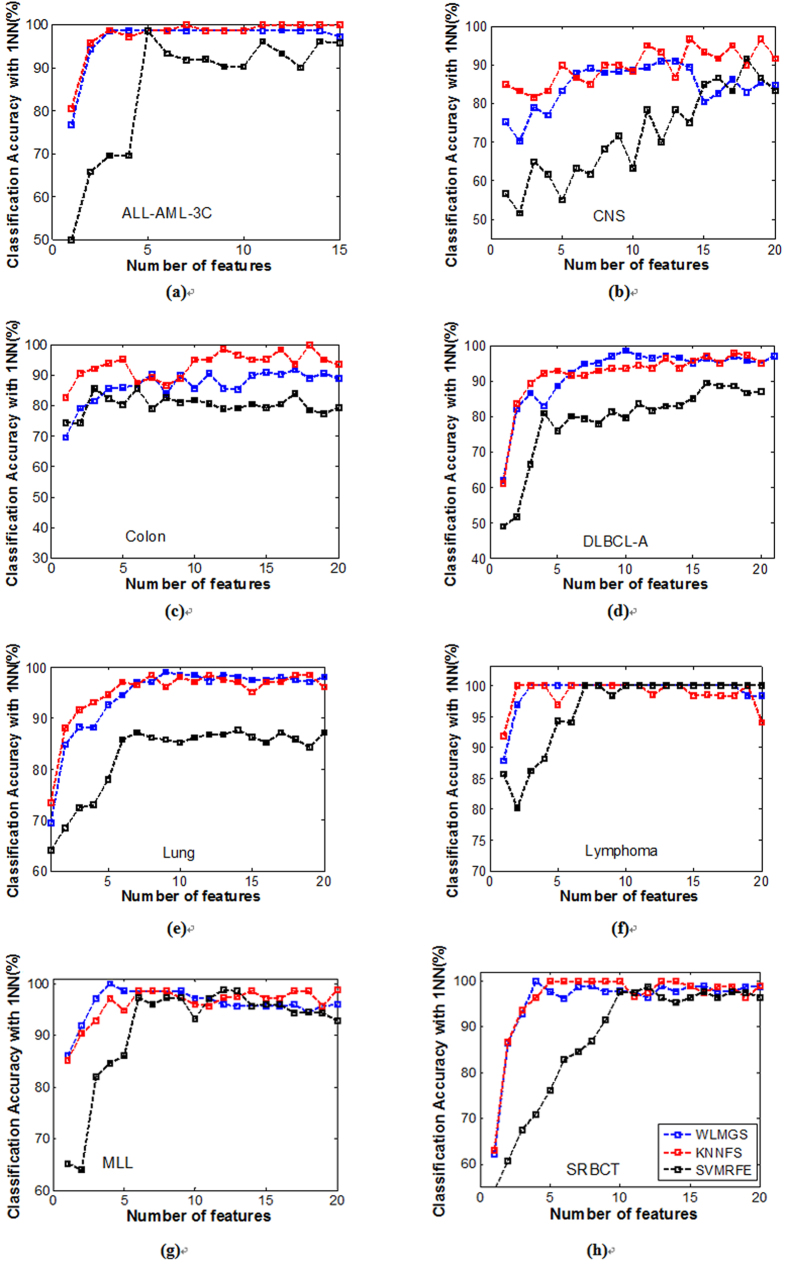
The average classification accuracy using 1NN classifier with respect to the subset of s features selected by different wrapped methods. For different methods, (**a**) is the classification accuracy in data ALL-AML-3c, (**b**) is the classification accuracy in data CNS, (**c**) is the classification accuracy in data Colon, (**d**) is the classification accuracy in data DLBCL-A, (**e**) is the classification accuracy in data Lung, (**f**) is the classification accuracy in data Lymphoma, (**g**) is the classification accuracy in data MLL, (**h**) is the classification accuracy in data SRBCT.

**Figure 4:**
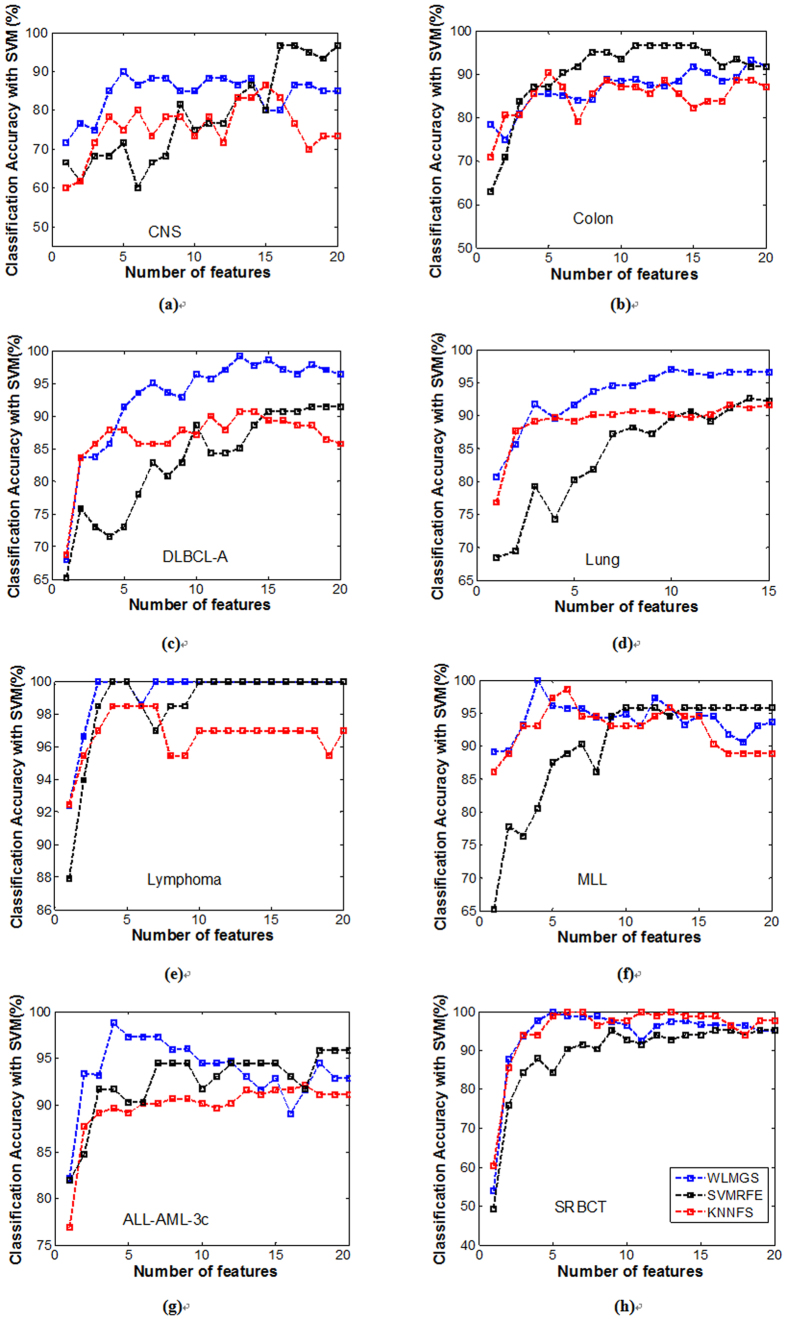
The average classification accuracy using SVM classifier with respect to the subset of s features selected by different wrapped methods. For different methods, (**a**) is the classification accuracy in data CNS, (**b**) is the classification accuracy in data Colon, (**c**) is the classification accuracy in data DLBCL-A, (**d**) is the classification accuracy in data Lung, (**e**) is the classification accuracy in data Lymphoma, (**f**) is the classification accuracy in data MLL, (**g**) is the classification accuracy in data ALL-AML-3c (**h**) is the classification accuracy in data SRBCT.

**Figure 5:**
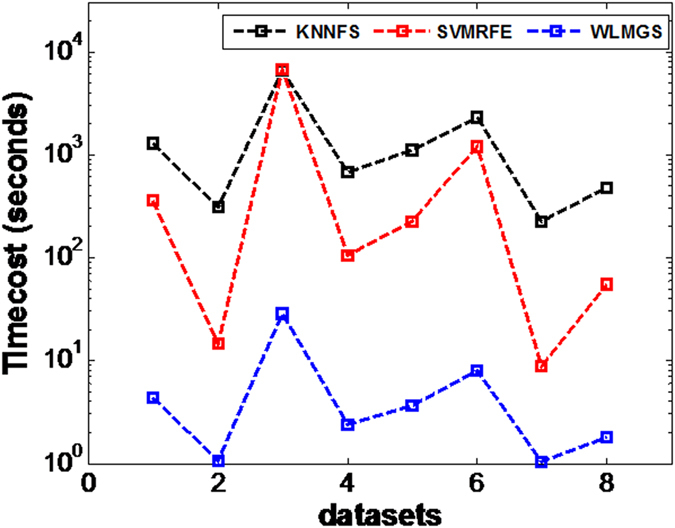
The average time cost in terms of Top 20 genes selected by our method and wrapped methods.

The comparison with the wrapped methods, such as the *SVMRFE* and the *KNNFS* has also been conducted in this study. As it is previously known, the wrapped algorithms depend on the classifier during the learning process. This results to greater classification accuracy, but this also requires a higher computational cost for a repeated training of classifiers other than the filter methods, such as the *SVMRFE* which depends on the SVM classifier, and the *KNNFS* which depends on the 1NN classifier. From Figs 3 and 4, the WLMGS method gets better results compared to the results of the *SVMRFE* and *KNNFS* methods in most cases. However, both the *SVMRFE* and *KNNFS* methods are more time-consuming than the *WLMGS* method as illustrated in Fig. 5. Therefore, for the purpose of efficiency, the filter methods are suggested to be applied in practice. This fact is verified from Figs 3 and 4, where the wrapped methods only perform well in the specified classifier, but worse in other classifiers. This means the wrapped methods possess the poor generalization ability. However, the *WLMGS* method achieves balanced results in both classifiers.

### Gene set enrichment analysis

In order to understand whether this study’s method is able to extract interactions with a biological meaning, the discriminatory gene subset selected by this method are analyzed by conducting the gene set enrichment analysis on the DAVID^41^ software (Database for Annotation, Visualization, and Integrated Discovery). DAVID is able to provide a comprehensive set of functional annotation tools for investigators to understand the biological meaning behind a large list of genes. The detailed information can be seen in ref. 41.

The top ten genes selected by using different methods are supplied into the DAVID website (https://david.ncifcrf.gov/home.jsp). The Functional Annotation Tool is utilized to achieve the Functional Annotation Clustering results (the Classification Stringency is set to High). The group Enrichment Score (ES) and the geometric mean (in -log scale) of the member’s p-values in a corresponding annotation cluster, is used to rank their biological significance. Thus, the top ranked annotation clusters most likely have consistently lower p-values for their annotation members. The larger the enrichment score, the more enriched is the gene subset.

For this present study’s method, the first value from the top annotation cluster having the largest ES, and the concerned terms having similar biological meanings are presented in Table 6. It is clearly seen that the genes selected by WLMGS are related to genes having cancer hallmarks (that is, genes that belong to the cancer-related Gene Ontology (GO) terms). This means that the gene subset selected by WLMGS is more enriched than the gene sets related to a biology process or a biology pathway.

To further verify the WLMGS’s effectiveness, the biological significance comparisons in terms of the enrichment score are achieved with *WLMGS* and the other methods as seen in Table 7. In general, the results in Table 7 indicate that the genes selected by *WLMGS* are more significant in the biological enrichment analysis.

## Discussion and Conclusion

As mentioned previously, the proposed approach in this study is able to capture different (small) gene subsets with a high prediction rate, which is important for further biological studies. More attention should be fixed on this approach to identify the biomarkers for the concerned cancer. Based on the weight local modularity, the proposed method in this study is able to explore the informative genes wherein the weighted sample graph has a small within-class distance and a large between-classes distance. Thus, the samples in the same class are possibly close to each other, and samples in different classes are likely far away from each other. This means that the samples are easily separated. From **Theorem 1,** the local cluster structure for the samples in a class also contribute to its higher *WLM*^*s*^, therefore, these specific genes is chosen to predict each cancer subtype. Furthermore, the method of this study is still efficient for the imbalanced dataset, because the weight local modularity considers the local cluster-connectivity and overcomes the global network dependency. The small sample cluster which includes only several samples, also helps to enhance the *WLM*^*s*^. Hence, the method of this study is preferred particularly for microarray datasets having a few samples. Therefore, this study’s method is able to select genes that have the best local and global structure preserving ability. Additionally, this study’s method is not affected by the noise sample points, where the small weights only have a minimal effect on *WLM*^*s*^. Lastly, the proposed method in this study can pick not only the discriminative genes individually, but also as a group. Both power genes as an individual and power genes as group but weak as individuals are explored for classification learning.

As previously discussed, there are two parameters in the method of this study which is used during the process of constructing the weighted sample graph and selecting genes, *e. g. λ*, *k.* The effect of these two parameters is studied in the following experiments.

**Figure 6:**
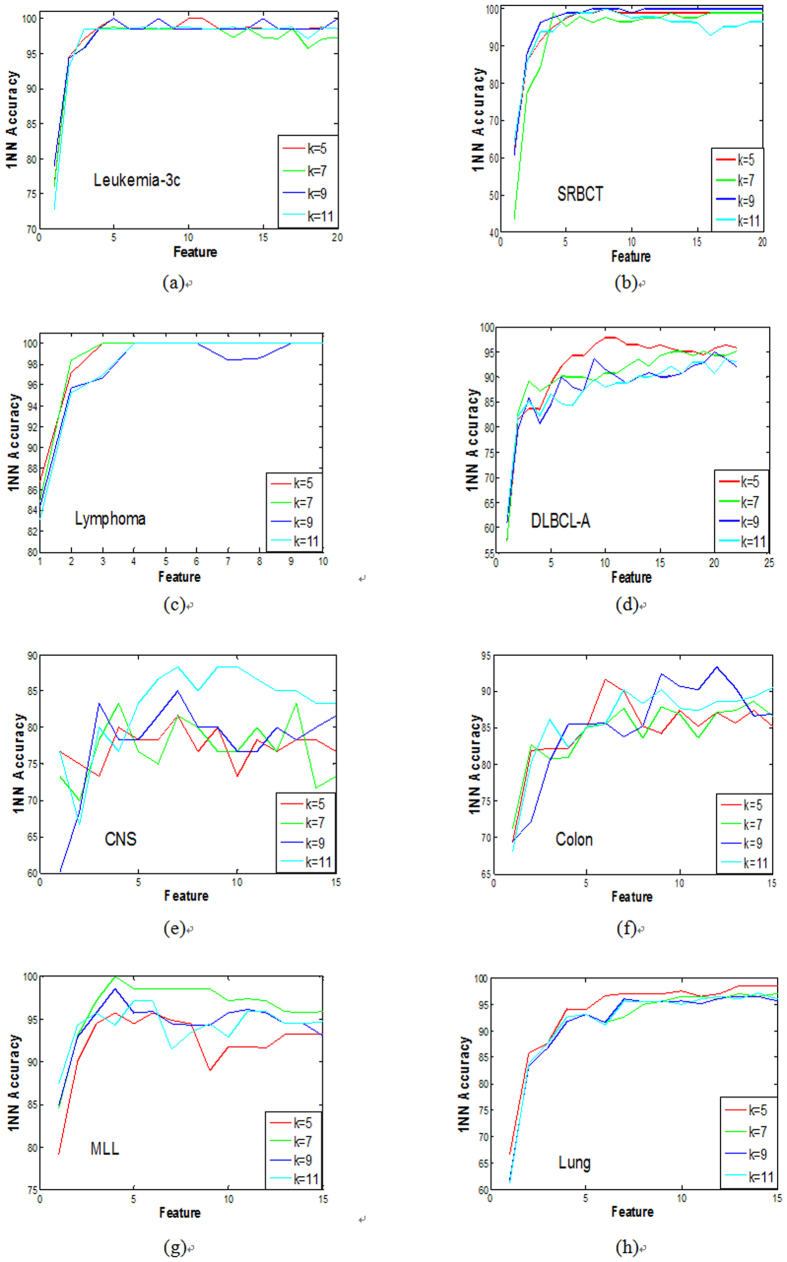
The average 1NN accuracy results on the different k for all datasets in our method. (**a**) is the classification accuracy on the different *k* for the data ALL-AML-3c, (**b**) is the classification accuracy on the different *k* for the data SRBCT, (**c**) is the classification accuracy on the different *k* for the data Lymphoma, (**d**) is the classification accuracy on the different *k* for the data DLBCL-A, (**e**) is the classification accuracy on the different *k* for the data CNS (**f**) is the classification accuracy on the different *k for the data Colon,* (**g**) is the classification accuracy on the different *k* for the data MLL, (**h**) is the classification accuracy on the different *k* for the data Lung.

The parameter *k* is the number of neighbors used in constructing the weighted sample graph. This determines the number of weight edges in a constructed samples graph, that is to say, a large *k* corresponds to a higher *w*_*k*_, *W*_*k*_, and *vice versa.* Figure 6 summarizes the average **1NN** accuracy results conducted ten times on all datasets using different values of *k* in this study’s method. It is clearly noted that *k* has an influence on the performance of some datasets, such as the **DLBCL_A** and the **CNS.** In fact, *k* is less than the number of samples in the smallest class. For most of the datasets, the *k* in {3–11} is more effective. In this study, different *k*’s are adapted to obtain a better performance for the different datasets.

The second parameter *λ* is the optimal number of selected genes. In practice, it is difficult to automatically determine the optimal number of selected genes. As previously discussed, the selection procedure should be stopped if the *WLM*^*s*^ is not increased greatly. As shown in Table 8, the incremental amount of *WLM*^*s*^ is proportional to its performance. The strategy in this study is that the iterative procedure will be terminated if the increment of *WLM*^*s*^ is lower than the very small value *θ*. From Fig. 6 and Table 8, it is good to determine the value of *λ* when the threshold *θ* of its difference is set to 0.02. As previously shown for all the datasets, the best performance is achieved within*λ*genes (less than 15 genes). Therefore, it is possible that the increment of *WLM*^*s*^ is the criterion for the termination of this study’s method, and this automatically determines the number of selected genes. The *λ* parameter was assigned with the value of 15 in this study.

### Conclusion

In this study, a new approach based on weight local modularity to gene subset selection is introduced. The sequential forward selection and the greedy search strategy are utilized to optimize the weight local modularity. Firstly, given a selected gene subset **S**, the candidate gene ***g*** is more informative if the increment of *WLM*^*s*^(*S* ∪ *g*) on the genes spaces *S* ∪ *g* is large. Furthermore, the number*λ* of the optimal genes is automatically determined by the incremental of *WLM*^*s*^. From the experiments, the *λ* is small enough to gain a better performance, which greatly reduces the time cost of this method. Additionally, the method in this study is effective for imbalance datasets. Experimental results demonstrate that the method in this study outperforms others. The gene subsets selected by this method are more enriched. The key reason is that weight local modularity captures both the local and global structures in the weight network. The weight local modularity provides the proposed method with the ability of not only finding a set of common genes differentiating all cancer types but also identifying the specific genes related to each type of cancers.

However, the computational cost of the proposed method is relatively higher than the others, even if the fast K-Nearest Neighbor Graph (K-NNG) algorithm is applied. The adaptive algorithm to choose the optimal *k* should be developed for different datasets.

In the future, recent technologies^42^ can be applied to further improve the accuracy and robustness of the predictors. It is known that tumor heterogeneity prevents the identification of robust cancer biomarkers. Some virtual data sets, called random microarray data sets (RDSs) were generated from the original data set. Next, distinct random gene sets (RGSs) were generated. A survival screen using each RGS against each RDS was performed. For each RDS, the gene sets and the survival screening P-value of which is <0.01, were achieved. The purpose of this is that a signature derived from one data set is transferable when applied to another data set. Also, an integrative network analysis of the gene signatures and the breast cancer driver-mutating genes in a protein interaction network, allowed the identification of several metastasis network modules. Each module contains the genes of one signature and their directly interacting partners that are cancer driver-mutating genes^42^. The approach^42^ might provide a framework for discovering robust and reproducible gene signatures for specific phenotypes, such as its clinical outcome, its drug response, or other disease features. The result provides a future research direction.

## Methods

### The definition of weighted local modularity in a complex network

**Figure 7:**
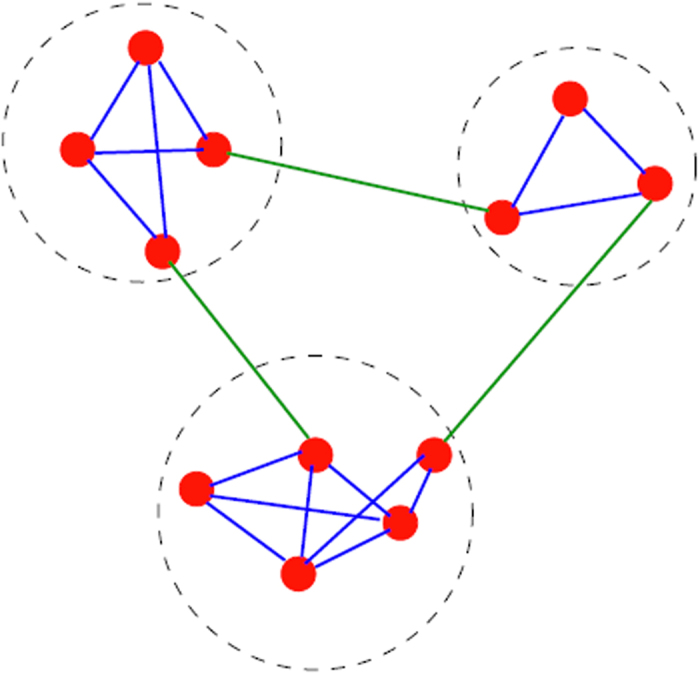
A simple graph with three local communities, enclosed by the dashed circles. Reprinted figure with permission from ref. 43.

A complex network is a graph (network) with non-trivial topological features—features that do not occur in simple networks such as lattices or random graphs, but often occur in graphs which are modelling real systems. Most of the real-world complex networks are composed of some communities or clusters within which they have close connections between nodes but have sparse connections between the various communities^43^, as seen in Fig. 7. Reliable algorithms^43^,^44^ are supposed to identify good partitions about clusters or communities, but the question remains on how clusterings are considered to be good or bad. In order to distinguish between ‘good’ and ‘bad’ partitions, the most accepted quality function is the modularity *Q* of Newman and Girvan^44^. The function is based on the idea that a random graph is not expected to have a cluster structure. In this way, the possible existence of clusters is revealed by the comparison between the actual density of edges in a subgraph and the density expected in the subgraph if the vertices of the graph were attached regardless of community structure. However, modularity *Q* optimization is widely criticized for its resolution limit^43^ because the modularity is a global measure. To overcome the problem, the localized modularity for the local structures of the undirection network has been introduced by considering the local cluster-connectivity^45^. In many complex networks most clusters are connected to only a small fraction of the remaining clusters, called the *local cluster connectivity.* From the view point of local connectivity, the local modularity (*LM*) is defined as follows:


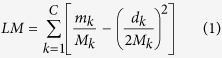


where *m*_*k*_ is the number of edges within the community *k* and *M*_*k*_ is the total number of edges in the community *k* and its first neighbor. *d*_*k*_ is the sum of the degrees of nodes in community *k*. *C* is the number of communities in the graph or the number of different classes for the samples in Eqs (4 and 5). The more locally connected clusters a network has, the higher is the *LM*.

However, as previously described, the *LM* is not able to evaluate the extra-class and intra-class distances because it is not related to the distance between nodes, which is not good for a classification problem. Therefore, in this study, the Weight Local Modularity *Q* (*WLM*) for the weighted graph is proposed and is defined by:


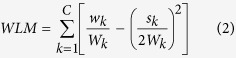


where *w*_*k*_ is the sum of the weights of the internal edges of community *k* and *W*_*k*_ is the total number of weights of the internal edges in community *k* and its first neighbor. *s*_*k*_ is the sum of the strengths of all the vertices in community *k*. The strength of a vertex is the sum of the weights of edges adjacent to the vertex. From Eq. 2, it can be concluded that a larger *WLM* corresponds to larger weights, (small distance in the same cluster) or to smaller weights (large distance between classes locally), which means that the nodes for different clusters are easily separated. Hence, these genes that minimize the within-cluster distance and maximize the between-cluster distance are preferred and get a higher weight^46^. Because of this this fact, this present study introduces a new local evaluation criterion for the gene subset using the weighted local modularity. The idea behind the method in this study is that the gene subset where the weighted sample graph has a higher. Having a higher *WLM* is more informative and the samples in the gene subset are classified accurately.

**Theorem 1.** Maximizing the 
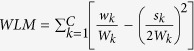
 is equivalent to simply maximizing the 
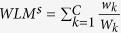
.

The proof can be seen in the Appendix section.Furthermore,


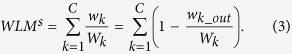


where *w*_*k*_*out*_ is the total weight of the first neighbors of the community *k* and *W*_*k*_ = *w*_*k*_ + *w*_*k*_*out*_.

It is worth noting that there is no difference on the classification performance between *WLM* and *WLM*^s^. In this paper, the simplified version *WLM*^s^ of WLM is used.

From **Theorem 1**, good local clusters are the ones with large internal weights (small internal distances) and small external weights (large external distances) as shown in Fig. 7. This result is consistent with the property of local cluster. Another advantage of *WLM*^*s*^ is that it is not affected by noisy samples. Noisy samples usually stay away from normal samples, which results in smaller weights in the weighted sample graph. From **Theorem 1**, smaller weights cause very little impact on *WLM*^*s*^. Therefore, in this study, the *WLM*^*s*^, which is a simple version of *WLM*, is adopted instead of LM and WLM.

A gene ***g*** is considered to be good if the *WLM*^*s*^ is increasing greatly while ***g***is accepted in the selected gene subset***gs***.

### Gene subset selection based on *WLM* ^*s*^

From the previous information on *WLM*^*S*^, the gene subset is preferred when the weighted sample graph has a higher *WLM*^*S*^.

### Weighted sample graph

Given a *m* × *n* microarray dataset (*m* corresponding to samples and *n* corresponding to genes), the gene subset **gs** which is selected from *n* genes is defined by: *gs* = {*g*_1_, *g*_2_, …, *g*_*n*1_}. The weighted sample graph *G*(*V*, *A*) in ***gs***is constructed as follows: there is a weighted edge *A*(*i*, *j*) between *v*_*i*_ and *v*_*j*_ if *v*_*i*_ ∈ *k* − *NN*(*v*_*j*_) or *v*_*j*_ ∈ *k* − *NN*(*v*_*i*_).

where *v*_*i*_ is the node *i* corresponding to *i*-th sample, *k* − *NN*(*v*_*i*_) is the *k-*neighborhoods set of node *i. A*(*i*, *j*) = exp(−*d*(*v*_*i*_, *v*_*j*_)), *d*(*v*_*i*_, *v*_*j*_) denotes the Euclidean distance between *v*_*i*_ and *v*_*j*_ : *d*(*v*_*i*_, *v*_*j*_) = ||*v*_*i*_ − *v*_*j*_||_2_, and in here, ||·||_2_ is the L2-norm. **A** is the affinity matrix. *k* is the predefined parameter. *k* does not take large values, and it ranges generally in {5–11}. This is discussed in section 5.3.

### Gene subset selection

This study’s method aims at identifying a gene subset where a weighted sample graph can achieve a larger weighted local modularity. To reduce the time complexity in the method of this study, the sequential forward during genes selection is adopted and the greedy search is utilized to optimize the *WLM*^*s*^. The proposed method namely *WLMGS* is illustrated in Algorithm 1.

*WLMGS* works in a straightforward way. Firstly, several relative parameters are initialized, *i.e.λ*, *k*, *gs* and the first gene*g*_1_ in *gs* where the weighted sample graph has the largest value is selected as the starting point of the search procedure. Sequentially, during each selection process, gene*g*with the largest *WLM*^*s*^ is selected to join ***gs*** from the ***G***. This iterative selection procedure will be terminated while the number of selected genes in ***gs*** is not less than the pre-specified threshold *λ*. In practice, the optimal *λ* is hard to determine due to the noisy genes that may increase the information amount of ***gs*** as they are selected. Because of this, the gene subset with the largest *WLM*^*s*^ may include noisy genes. To alleviate this problem, this study presents an alternative way to assign *λ* with an appropriate value wherein the iterative procedure will be terminated if the difference of *WLM*^*s*^(*gs*_*i*+1_) with *WLM*^*s*^(*gs*_*i*_) is lower than a very small value *θ*. The gene *g*_*i*+1_brings minimal information to the selected genes in ***gs*** and the *WLM*^*s*^(*gs*_*i*+1_) increases lightly after it has been picked into ***gs,*** where *gs*_*i*_ and *gs*_*i*+1_ are the selected gene subset in the *i*-th and (*i* + 1)-th iterations, respectively. As it is known, the selection procedure should be stopped if the information embodied by ***gs*** does not increase greatly^47^.

Additionally, the genes selected based on *WLM*^*s*^ have a large *relevant independency (RI)* which contributes to a better classification accuracy. The samples in the selected genes can be easily separated. The gene subset as a group selected by this study’s method has a strong relevance with its class label. Therefore, method of this study can address the problem of redundancy among genes.

In *WLMGS*, the most time-consuming step is the construction of the weighted sample graph iteratively. The total is about *ο*(*λnm*^2^). In this study, the fast K-Nearest Neighbor Graph (K-NNG) construction method^48^,^49^ is applied to the construction of the weighted sample graph, which reduces the time complexity from *ο*(*λnm*^2^) to *ο*(*λnm*^1 ^14).

Algorithm 1. *WLMGS*: *WLM* based Gene Selection


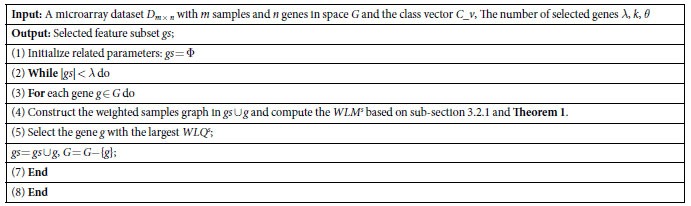


*λ*: the number of selected genes.

*k*: the number of Nearest Neighbor in constructing the weighted sample graph.

*θ*: the difference of *WLM*^*s*^(*gs*_*i*+1_) with *WLM*^*s*^(*gs*_*i*_).

### Justification of *WLMGS* based on k-means cluster

In this section, the proposed feature evaluation criterion based on the weight local modularity is demonstrated by the theory of k-means cluster.

The k-means cluster^50^ is the most well-known clustering algorithm. This algorithm iteratively attempts to address the following objective: given a set of points in a Euclidean space and a positive integer *p* (the number of clusters), the points are split into *p* clusters so that the total sum of the Euclidean distances of each point to its nearest cluster center is minimized, and is defined as follows:


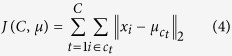


Here, *x*_*i*_ and 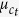 is respectively the *i*-th sample point and its nearest cluster center, ||·||_2_ is the L2-norm.

In the weighted feature approache by k-means, the features that minimize the within-cluster distance and simultaneously maximize between-cluster distance are preferred. It can be confirmed clearly in **Theorem 2** that the genes having a higher *WLM*^*s*^ in this study’s method is able to simultaneously minimize the within-cluster distance and the maximize between-cluster distance.

According to **Theorem 1,** 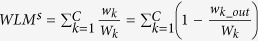, making *WLM*^*s*^ higher is equivalent to maximizing the inner weight *w*_*k*_ and in minimizing the outer weight *w*_*k*_*out*_, that is to say, each community of the weighted sample graph has a larger *w*_*k*_ and a smaller *w*_*k*_*out*_.

Given the selected gene subset **S,** the candidate gene ***g,*** and the weighted sample graph in*S* ∪ *g*genes space, then:


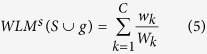


The gene ***g*** with a larger increment of *WLM*^*s*^(*S* ∪ *g*) is more discriminative.

According to Eqs (3, 4, 5), **Theorem 2** is verified as follows:

**Theorem 2.** Maximizing the *WLM*^*s*^ is equivalent to minimizing the k-means cluster objective *J*(*C*, *μ*).

The proof is seen in the Appendix section.

Therefore, *J*(*C*, *μ*) in *S* ∪ *g* is minimizing while the *WLM*^*s*^of WSG in *S* ∪ *g* gets a higher value. This finding indicates that genes selected by this study’s method are able to minimize the within-cluster distance (large *w*_*k*_) and maximize the between-cluster distance (small *w*_*k*_*out*_). It is considered that the gene subset with a higher *WLM*^*s*^ is more relevant within its class label, which not only minimizes the within-cluster distance, but also maximizes the between-cluster distance.

## Additional Information

**How to cite this article**: Zhao, G. and Wu, Y. Feature subset Selection for Cancer Classification Using Weight Local Modularity. *Sci. Rep.* **6**, 34759; doi: 10.1038/srep34759 (2016).

## Supplementary Material

Supplementary Information

## Figures and Tables

**Table 1:** Description of the datasets.

Number	Data set	Genes	Samples	Classes
1	ALL-AML-3C	7129	72	3
2	DLBCL_A	661	141	3
3	SRBCT	2308	83	4
4	MLL	12582	72	3
5	CNS	7129	60	2
6	Lymphoma	4026	66	3
7	Colon	2000	62	2
8	Lung	12600	203	5

**Table 2:** Comparisons of the best results between *WLMGS* and others with **1NN** classifier.

Classifiers	*1NN*						
Dataset		*mRMR*	*MIFS_U*	*CMIM*	*Relief*	*CMQFS*	*WLMGS*
ALL-AML-3C	|#G|	**3**	**3**	**3**	15	28	**3**
*k* = 7	*ACC*	**98.57**	**98.57**	**98.57**	94.46	98.61	**98.57**
DLBCL_A	|#G|	22	14	3	22	29.35	**10**
*k* = 5	*ACC*	95.71	90.91	85.76	93	93.91	**98.62**
SRBCT	|#G|	12	29	16	28	25	4
*k* = 9	*ACC*	**100**	98.75	**100**	100	100	**100**
MLL	|#G|	27	16	2	7	27	**4**
*k* = 7	*ACC*	**100**	98.57	95.89	94.46	100	**100**
CNS	|#G|	9	3	**2**	30	3	12
*k* = 11	*ACC*	73.66	78	85.01	84.12	84.5	**91**
Lymphoma	|#G|	5	5	8	8	21	**3**
*k = *7	*ACC*	**100**	**100**	98.33	**100**	**100**	**100**
Colon	|#G|	12	19	17	4	13	17
*k* = 9	*ACC*	90.23	90.23	90.71	75.95	90.76	**91.67**
Lung	|#G|	28	13	26	30	27	9
*k* = 5	*ACC*	93.61	94.59	95.11	93.04	95.15	**99.02**

Note: |#G|: average number of genes; ACC: average classification accuracy (%); T: average time (s) in selected 15 genes.

**Table 3:** Comparisons of the best results between *WLMGS* and others with **SVM** classifier.

Classifiers		SVM					
Dataset		mRMR	MIFS_U	CMIM	Relief	CMQFS	WLMGS
ALL-AML-3C	|#G|	**3**	3	11	33	27	4
k = 7	ACC	98.57	98.57	97.32	96.07	98.51	**98.75**
DLBCL_A	|#G|	16	16	31	30	29	**13**
k = 5	ACC	98.66	91.47	97.23	93.67	95.81	**99.28**
SRBCT	|#G|	12	13	16	28	7	**5**
k = 9	ACC	**100**	95.27	**100**	100	100	**100**
MLL	|#G|	16	16	17	20	26	**4**
k = 7	ACC	**100**	**100**	98.75	85.17	98.61	**100**
CNS	|#G|	8	25	7	30	26	5
k = 11	ACC	75	76.67	85	75.12	82.16	90
Lymphoma	|#G|	5	5	17	28	22	**3**
k = 7	ACC	**100**	**100**	**100**	92.38	99.85	**100**
Colon	|#G|	11	22	16	25	23	19
k = 9	ACC	89.28	91.67	81.74	80.71	87.95	93.33
Lung	|#G|	11	14	15	15	15	10
k = 5	ACC	94.54	94.59	95.52	81.33	92.71	**97.07**

Note: |#G|: average number of genes; ACC: average classification accuracy (%); T: average time (s) in selected 15 genes.

**Table 4:** *p*-Values between *WLMGS* and other methods about *ACC* and |#G| with 1NN.

methods	ACC-p	|#G|-p
WLMGS vs. mRMR	0.028	0.021
WLMGS vs. MIFS_U	0.013	0.019
WLMGS vs. CMIM	0.004	0.013
WLMGS vs. Relief	0.000	0.000
WLMGS vs. CMQFS	0.000	0.000

**Table 5:** *p*-Values between *WLMGS* and other methods about *ACC* and |#G| with SVM.

methods	ACC-p	|#G|-p
WLMGS vs. mRMR	0.044	0.046
WLMGS vs. MIFS_U	0.029	0.014
WLMGS vs. CMIM	0.037	0.004
WLMGS vs. Relief	0.002	0.000
WLMGS vs. CMQFS	0.000	0.000

**Table 6:** The enrichment analysis results about annotation cluster by DAVID in Top ten genes selected by *WLMGS*.

Dataset	Annotation Cluster	Enrichment Score
ALL-AML_3c	GO:0002521~leukocyte differentiation, GO:0030097~hemopoiesis, GO:0048534~hemopoietic or lymphoid organ development, GO:0002520~immune system development	2.07
CNS	GO:0005261~cation channel activity, GO:0046873~metal ion transmembrane transporter activity, GO:0005216~ion channel activity, GO:0022838~substrate specific channel activity, GO:0015267~channel activity, GO:0022803~passive transmembrane transporter activity, GO:0030001~metal ion transport, GO:0006812~cation transport, GO:0006811~ion transport, SP_PIR_KEYWORDS~disease mutation, UP_SEQ_FEATURE~sequence variant, SP_PIR_KEYWORDS~polymorphism	2.31
MLL	GO:0030528~transcription regulator activity, GO:0006350~transcription, GO:0045449~regulation of transcription, SP_PIR_KEYWORDS~Transcription	0.59
Lung	GO:0005615~extracellular-space, GO:0044421~extracellular region part, GO:0005576~extracellular region	1.78

**Table 7:** The enrichment score results by DAVID in Top ten genes selected by different methods.

	ALL-AML_3c	CNS	MLL	Lung
*mRMR*	2.19	1.91	0.59	0.65
*MIFS_U*	0.33	0.65	**2.01**	0.31
*CMIM*	**5.19**	0.36	0.41	0.2
*Relief*	0.15	0.21	0.23	0.15
*CMQFS*	1.79	2.05	0.55	1.54
*WLMGS*	2.07	**2.31**	0.59	**1.78**

The larger the enrichment score, the more enriched the genes subset.

**Table 8:** The increment of *WLM* ^*s*^with selected genes.

Dataset	Increment of *WLM*^*s*^during selection	AN_B
ALL-AML-3C	0.72, 0.32, 0.24, 0.05, 0.00, 0.00, 0.00, 0.00, 0.00, 0.00, 0.00, 0.00, 0.00, 0.00	4.35
DLBCL_A	0.52, 0.17, 0.27, 0.16, 0.14, 0.11, 0.07, 0.09, 0.02, 0.02, 0.00, 0.00, 0.00, 0.00	9.73
SRBCT	0.83, 0.82, 0.40, 0.14, 0.12, 0.02, 0.00, 0.00, 0.00, 0.00, 0.00, 0.00, 0.00, 0.00	7.56
MLL	0.45, 0.30, 0.11, 0.03, 0.00, −0.00, −0.00, 0.01, 0.04, 0.01, 0.00, 0.00, 0.00, 0.00	4.72
CNS	0.13, 0.17, 0.12, 0.14, 0.07, 0.08, 0.05, 0.09, 0.05, 0.01, 0.02, 0.02, 0.01, 0.00	7.23
Lymphoma	0.58, 0.14, 0.01, 0.00, 0.00, 0.00, 0.00, 0.00, 0.00, 0.00, 0.00, 0.00, 0.00, 0.00	3.00
Colon	0.13, 0.06, 0.15, 0.13, 0.13, 0.07, 0.05, 0.05, 0.01, 0.00, 0.00, 0.00, 0.00, 0.00	12.73
Lung	1.03, 0.63, 0.34, 0.22, 0.30, 0.20, 0.06, 0.08, 0.04, 0.03, 0.02, 0.00, 0.00, 0.00	13.29

Note: AN_B: the average number of selected genes while the best result is obtained.

## References

[b1] José E. A., Garć ıa. N., Jourdan L. & Talbi E. G. Gene selection in cancer classification using PSO/SVM and GA/SVM hybrid algorithms. IEEE C. Evol. Computat. 9, 284–290 (2007).

[b2] Derrac J., Cornelis C., García S. & Herrera F. Enhancing evolutionary instance selection algorithms by means of fuzzy rough set based feature selection. Information Sciences 186, 73–92 (2012).

[b3] Sun X., Liu Y. H., Wei D. & Xu M. T. Selection of interdependent genes via dynamic relevance analysis for cancer diagnosis. J. Biomed. Inform. 46, 252–258 (2013). 23124059 10.1016/j.jbi.2012.10.004

[b4] Guyon I., Weston J., Barnhill S. & Vapnik V. Gene selection for cancer classification using suppor tvector machines. Mach. Learn. 46, 389–422 (2002).

[b5] Saeys1 Y., Inza Iñ & Larrañaga P. A review of feature selection techniques in bioinformatics. Bioinformatics 23, 2507–2517 (2007). 17720704 10.1093/bioinformatics/btm344

[b6] Yang P. Y., Yang Y. H., Zhou B. B. & Zomaya A. Y. A review of Ensemble Methods in Bioinformatics. Current Bioinformatics. 5, 296–308 (2010).

[b7] Kohavi R. & John G. H. Wrappers for feature subset selection. Artificial Intelligence. 97, 273–324 (1997).

[b8] Jafari P. & Azuaje F. An assessment of recently published gene expression data analyses: reporting experimental design and statistical factors. BMC Med. Inform. Decis. Mak. 6, 27 (2006). 16790051 10.1186/1472-6947-6-27 PMC1523197

[b9] Thomas J. G., Olson J. M. & Tapscott S. J. An efficient and robust statistical modeling approach to discover differentially expressed genes using genomic expression profiles. Genome Res. 11, 1227–1236 (2001). 11435405 10.1101/gr.165101 PMC311075

[b10] Rainer B. L., Patrick A., Anna A. & Pawel H. Rank products: a simple, yet powerful, new method to detect differentially regulated genes in replicated microarray experiments. FEBS Lett. 573, 83–92 (2004). 15327980 10.1016/j.febslet.2004.07.055

[b11] Thomas J. G. An Efficient and Robust Statistical Modeling Approach to Discover Differentially Expressed Genes Using Genomic Expression Profiles. Genome Res. 11, 1227–1236 (2001). 11435405 10.1101/gr.165101 PMC311075

[b12] Dudoit S. Statistical Methods for Identifying Differentially Expressed Genes in Replicated cDNA Microarray Experiments. Statistica. Sinica. 12, 111–139 (2002).

[b13] Long A. D. Improved Statistical Inference from DNA Microarray Data Using Analysis of Variance and A Bayesian Statistical Framework. J. Biolog. Chemis. 276, 19937–19944 (2001). 10.1074/jbc.M010192200 11259426

[b14] Chuang L. Y., Yang C. H. & Li J. C. A hybrid BPSO-CGA approach for gene selection and classification of microarray data. J. Comput. Biol. 19, 1–14 (2011). 21210743 10.1089/cmb.2010.0064 PMC3244808

[b15] Wang Y. . Gene selection from microarray data for cancer classification–a machine learning approach. Comput. Biol. Chem. 29, 37–46 (2005). 15680584 10.1016/j.compbiolchem.2004.11.001

[b16] Gevaert O., De Smet F., Timmerman D. & Bart L. R. Predicting the prognosis of breast cancer by integrating clinical and microarray data with Bayesian networks. Bioinformatics. 22, 184–190 (2006). 10.1093/bioinformatics/btl230 16873470

[b17] Peng H., Long F. & Ding C. Feature selection based on mutual information: criteria of max-dependency, max-relevance, and min-redundancy. IEEE Trans. Pattern. Anal. Mach. Intell. 27, 1226–1238 (2005). 16119262 10.1109/TPAMI.2005.159

[b18] Battiti R. Using mutual information for selecting features in supervised neutral net learning, IEEE Trans. Neu. Netw. 5, 537–550 (1994). 10.1109/72.298224 18267827

[b19] Kwak N. & Choi C. H. Input feature selection for classification problems. Ieee t. Neural. Networ 13, 143–159 (2002). 10.1109/72.977291 18244416

[b20] Fleuret F. Fast binary feature selection with conditional mutual information. J.Mach. Learn. Res. 5, 1531–1555 (2004).

[b21] Yusta S. C. Different metaheuristic strategies to solve the feature selection problem. Pattern. Recogn. Lett. 30, 525–534 (2009).

[b22] Bermejo P., Gámez J. A. & Puerta J. M. A GRASP algorithm for fast hybrid (filter-wrapper) feature subset selection in high-dimensional datasets. Pattern. Recogn. Lett. 32, 701–711 (2011).

[b23] Evers L. & Messow C. M. Sparse kernel methods for high-dimensional survival data. Bioinformatics. 24, 1632–1638 (2008). 18515276 10.1093/bioinformatics/btn253

[b24] Saeys Y. . Feature selection for splice site prediction: a new method using EDA-based feature ranking. BMC Bioinforma 5, 64 (2004). 10.1186/1471-2105-5-64 PMC421631 15154966

[b25] Zhu Y., Shen X. & Pan W. Network-based support vector machine for classification of microarray samples. BMC Bioinforma. 10, 21–25 (2009). 10.1186/1471-2105-10-S1-S21 PMC2648796 19208121

[b26] Li L. . A robust hybrid between genetic algorithm and support vector machine for extracting an optimal feature gene subset. Genomics. 8, 516–523 (2005). 10.1016/j.ygeno.2004.09.007 15607418

[b27] Li L. . Gene assessment and sample classification for gene expression data using a genetic algorithm/k-nearest neighbor method. Comb. Chem. High. T. Scr. 4, 727–739 (2001). 10.2174/1386207013330733 11894805

[b28] Zhao G. D., Wu Y., Ren Y. F. & Zhu M. EAMCD: an efficient algorithm based on minimum coupling distance for community identification in complex networks. Eur. Phys. J. B. 86, 14 (2013).

[b29] Christophe A. & McLachlan G. J. Selection bias in gene extraction on the basis of microarray gene-expression data. Proceedings of the National Academy of Sciences. 99, 6562–6566 (2002). 10.1073/pnas.102102699 PMC124442 11983868

[b30] Zhao G. D. . Effective feature selection using feature vector graph for classification. Neurocomp. 151, 376–389 (2015).

[b31] Kira K. & Rendell L. A practical approach to feature selection. Proc. 9th International Workshop on *Machine Learning*, 249-256 (1992).

[b32] Xue Y. M. . A comparison between two KNN based feature selection algorithms. Electronic Design Engineering, 24, 19–22 (2016).

[b33] Hoshida Y. . Subclass Mapping: Identifying Common Subtypes in Independent DiseaseDataSets. PloS ONE. 2, 11 (2007). 10.1371/journal.pone.0001195 PMC2065909 18030330

[b34] Li T., Zhang C. & Ogihara M. A comparative study of feature selection and multiclass classication methods for tissue classication based on gene expression. Bioinformatics 20, 2429–2437 (2004). 15087314 10.1093/bioinformatics/bth267

[b35] Li J. & Liu H. Kent Ridge Biomedical Data Set Repository, Http://sdmclit.org.sg/GEDatasets, 2002.

[b36] Sakar C. O. A feature selection method based on kernel canonical correlation analysis and the minimum Redundancy-Maximum Relevance filter method. Exp. Syst. with Appl. 39, 3432–3437 (2012).

[b37] Kursun O., Sakar C. O., Favorov O. N. & Aydin F. Using covariates for improving the minimum redundancy maximum relevance feature selection method. Tur. *J. Elec. Eng. & Comp.* Sci. 18, 975–989 (2010).

[b38] Brown G., Pocock A., Zhao M. J. & Luj´an M. Conditional Likelihood Maximisation: A Unifying Framework for Information Theoretic Feature Selection. J. Mach. Learn. Res. 13, 27–66 (2012).

[b39] He Q., Wu C., Chen D. & Zhao S. Fuzzy rough set based attribute reduction for information systems with fuzzy decisions. Knowl-based Syst. 24, 689–696 (2011).

[b40] Chen Y., Miao D., Wang R. & Wu K. A rough set approach to feature selection based on power set tree. Knowl-based Syst. 24, 275–281 (2011).

[b41] Dennis G. J. . DAVID: Database for Annotation, Visualization, and Integrated Discovery. Genome. Biol. 4, 3 (2003). PMC193660 12734009

[b42] Li J. . Identification of high-quality cancer prognostic markers and metastasis network modules. Nat. Commun. 1, 34doi: 10.1038/1033 (2010). 20975711 PMC2972666

[b43] Fortunato S. Community detection in graphs. Phys. Rep. 486, 75–174 (2010).

[b44] Newman M. E. J. & Girvan M. Finding and evaluating community structure in networks. Phys. Rev. E. 69, 026113 (2004). 10.1103/PhysRevE.69.026113 14995526

[b45] Muff S., Rao F. & Caflisch A. Local modularity measure for network clusterizations. Phys. Rev. E. 72, 056107 (2005). 10.1103/PhysRevE.72.056107 16383688

[b46] Alelyani S., Tang J. & Liu H. Feature Selection for Clustering: A Review. In: Aggarwal C., Reddy C. (eds) Data Clustering: Algorithms and Applications. CRC Press (2013).

[b47] Ambroise, Christophe & Geoffrey J. McLachlan. Selection bias in gene extraction on the basis of microarray gene-expression data. Proceedings of the National Academy of Sciences 99(10), 6562–6566 (2002). 10.1073/pnas.102102699 PMC124442 11983868

[b48] Garcia V., Debreuve E. & Barlaud M. Fast k nearest neighbor search using GPU. Proc. IEEE Conf. Comput. Vision and Pattern Recognition : *Comput. Vision* on GPU, Anchorage, Alaska, USA. *IEEE Computer Society press*. 24–26 (2008).

[b49] Wei D., Moses C. & Li K. Efficient K-Nearest Neighbor Graph Construction for Generic Similarity Measures. International *World Wide Web* Conference Committee (IW3C2), Hyderabad, *India. IEEE press.* March 28–April 1 (2011).

[b50] Boutsidis C., Drineas P. & Mahoney M. W. Unsupervised feature selection for the k-means clustering problem. Adv. Neural Inf. Process Syst. 6, 153–161 (2009).

