# Supplementary material for: Feature Subset Selection for Cancer Classification Using Weight Local Modularity
Source: Sci Rep. 2016 Oct 5;6:34759. doi: 10.1038/srep34759 (PMC5050509; doi:10.1038/srep34759)
Supplement: Supplementary Information [file srep34759-s1.doc]

**Feature subset Selection for Cancer Classification**

**Using Weight Local Modularity**

**Guodong Zhao &Yan Wu^*^**

**Appendix**

**Theorem 1.** Maximizing the is equivalent to maximizing the simply.

The proof can be seen in Appendix 1.

**Proof:**  can be expanded as following:

()

()

which proves **Theorem 1**.

**Theorem 2.** Maximizing the is equivalent to minimize the k-means cluster objective.

**Proof:** maximizing minimizing

and

(*n* is the number of nodes in graph)

So, maximizing is equivalent to minimize .

and

where is the number of samples in class . and is respectively the *i*-th sample point and its nearest cluster center .

Hence, the k-means cluster function is being minimized while is being minimized, being maximized.
